# Effect of swimming initiation period and continuation frequency on motor competence development in children aged up to 3 years: the Japan environment and children’s study

**DOI:** 10.1186/s13102-024-00980-9

**Published:** 2024-09-17

**Authors:** Hirohisa Kano, Takeshi Ebara, Taro Matsuki, Hazuki Tamada, Yasuyuki Yamada, Sayaka Kato, Kayo Kaneko, Kazuki Matsuzaki, Hirotaka Sato, Kyoko Minato, Mayumi Sugiura-Ogasawara, Shinji Saitoh, Michihiro Kamijima

**Affiliations:** 1 Department of Occupational and Environmental Health, Graduate School of Medical Sciences, Nagoya City University, 1 Kawasumi, Mizuho-cho, Mizuho-ku, Nagoya, Aichi 467-0001 Japan; 2 School of Health and Sport Sciences, Chukyo University, 101 Tokodachi, Kaizu-cho, Toyota, Aichi 470- 0393 Japan; 3 Institute of Industrial Ecological Sciences, the University of Occupational and Environmental Health, 1-1 Iseigaoka, Yahatanishi-ku, Kitakyushu, Fukuoka 807-8555 Japan; 4 Faculty of Health Sciences, Hiroshima International University, 555-36 Kurosegakuendai, Higashihiroshima, Hiroshima 739-2695 Japan; 5 Faculty of Food and Health Sciences, Aichi Shukutoku University, 2-9 Katahira, Nagakute, Aichi 480-1197 Japan; 6 Graduate School of Health and Sports Science, Juntendo University, 1-1 Hiraka-gakuendai, Inzai, Chiba 270-1695 Japan; 7 Faculty of Health Sciences, Japan University of Health Sciences, 1961-2 Satte, Satte, Saitama 340-0113 Japan; 8 Department of Obstetrics and Gynecology, Graduate School of Medical Sciences, Nagoya City University, 1 Kawasumi, Mizuho-cho, Mizuho-ku, Nagoya, Aichi 467-0001 Japan; 9 Department of Pediatrics and Neonatology, Graduate School of Medical Sciences, Nagoya City University, 1 Kawasumi, Mizuho-cho, Mizuho-ku, Nagoya, Aichi 467-0001 Japan

**Keywords:** Baby swimming, Gross motor, Fine motor, The Japan Environment and Children’s study, Large-scale prospective cohort study, Motor development

## Abstract

**Background:**

Although involvement of toddlers in swimming activities has increased recently, information regarding the impact of swimming during toddlerhood on subsequent child motor competence development is scarce. This study aimed to determine how swimming experience, particularly the timing of initiation and the continuity of swimming activities up to the age of 3 years, affects motor competence development.

**Methods:**

This prospective cohort study included data on children aged 1.5 and 3 years (100,286 mother–child pairs) from the Japan Environment and Children’s Study. The outcomes measured were gross and fine motor function, using the Japanese version of the Ages and Stages Questionnaire (Third edition). We assessed how these functions correlated with the continuous pattern of swimming pool use frequency from age 1 up to 3 years.

**Results:**

The group that used a swimming pool once a month or more from age 1–1.5 years but stopped from age 2–3 years showed consistently significant negative associations with gross motor development delay (minimum adjusted odds ratio [aOR]: 0.66, 95% confidence interval [CI]: 0.60–0.73) and fine motor development delay (minimum aOR: 0.66, 95% CI: 0.58–0.76). The group that continued swimming once a month or more from age 1–3 years showed consistently significant negative associations with gross motor development delay (minimum aOR: 0.64, 95% CI: 0.54–0.75) and fine motor development delay (minimum aOR: 0.42, 95% CI: 0.31–0.55).

**Conclusions:**

These results suggest that swimming experience starting around age 1 year is positively associated with gross and fine motor function development. The beneficial impact on gross motor function persisted from age 1–3 years. In contrast, the effects on fine motor function were not evident until age ≥ 2.5 years after starting swimming at approximately age 1 year. These findings underscore the potential benefits of early swimming experiences in enhancing overall motor skills development during early childhood.

## Background

Swimming is one of the most popular physical activities among children worldwide [1]. Recently, there has been a significant increase in the participation of young children in swimming (hereafter referred to as “baby swimming”) [2]. Originating in the United States and introduced in Japan during the 1970s, baby swimming has evolved into an underwater exercise program aimed at promoting growth and development [3, 4]. The target age for baby swimming typically ranges from approximately 6 months to 3 years. Starting at approximately 6 months allows children to have gained sufficient neck and trunk control, and the ability to sit independently, while also allowing their bodies to gain stability, making parents comfortable holding them in water. The baby swimming program generally concludes around age 3 years, as children begin to develop independence and social skills, transitioning to classes for older children (age ≥ 3 years) who participate without their parents [4, 5].

Generally, baby swimming is believed to contribute to improving swimming skills, preventing water accidents, and improving sensory function and motor competence [2], owing to the significant development of the nervous system during early childhood. Additionally, regular swimming during early childhood is expected to influence motor competence, defined here as the overall ability to perform various motor tasks effectively and efficiently, including swimming behavior [6, 7], coordination ability [8], object control skills [9], as well as gross and fine motor function [10]. To ensure consistency in terminology, this study will use “motor competence” when referring to motor skills. However, despite the growing expectations for motor competence development through swimming experience, limited studies have investigated the relationship between baby swimming and motor competence in children aged approximately 6 months to 3 years.

Several previous studies have demonstrated that swimming experience up to 3 years of age affects motor competence development. For instance, baby swimming experience was found to affect motor competence, including ball skills and static balance, at age 4 years [11]. Moreover, regular interventions in baby swimming have been shown to have positively impact the development of gross and fine motor function [12–14]. These studies are significant as they evaluated the positive effects of swimming on motor competence both before and after the implementation of swimming programs. However, their limitation include small sample sizes (< 40 participants per study), which restricts the generalizability of their findings. Specifically, large-scale epidemiological studies that have longitudinally examined the effects of swimming experience up to the age of 3 years on the development of motor competence are currently lacking.

Therefore, using a longitudinal design, the present study aimed to investigate how the presence or absence of regular swimming experience up to the age of 3 years, particularly focusing on the timing of initiation and continuity of swimming practice, influences motor competence.

## Methods

### Study design and participants

**Fig. 1:**
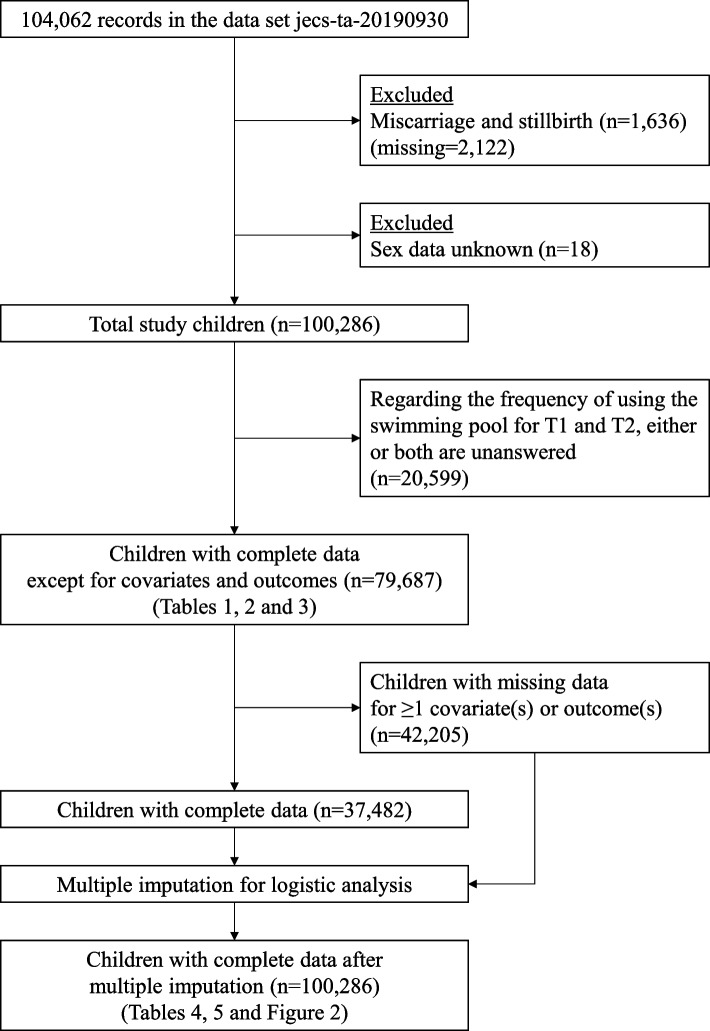
Flow chart of study participants

This longitudinal study enrolled pregnant women from the Japan Environment and Children’s Study (JECS), a large prospective cohort study investigating environmental factors affecting the health and development of children [15–17]. Pregnant women were recruited between January 2011 and March 2014. The inclusion criteria were (1) pregnant women residing in areas covered by each of the 15 Regional Centers at the time of recruitment, (2) expected delivery after August 1, 2011, and (3) proficiency in Japanese to respond to self-administered questionnaires. This study utilized the jecs-ta-20190930 dataset, which included 104,062 fetal records and was released under restrictions to relevant parties in October 2019. Women completed questionnaires regularly from pregnancy until their child reached 3 years of age. We excluded fetal records involving miscarriages and stillbirths (*n* = 1,636), those lacking birth status data (*n* = 2,122), and those without sex data (*n* = 18), and data from 100,286 mother-child pairs were analyzed. Of those included, 62,804 children had missing data for one or more variables, and 37,482 children had complete data for all variables. Missing values were treated using multiple imputations, assuming they were missing at random (Fig. 1).

The JECS protocol received approval from the Institutional Review Board of the Ministry of Environment on Epidemiological Studies and the Ethics Committees of all participating institutions. All participants provided written informed consent.

### Variables

#### Exposures

The questionnaire data on children’s pool use frequency were collected twice between birth and age 3 years. The first collection (Time 1; T1) occurred when the child was 1.5 years old, and the second collection (Time 2; T2) was conducted when the child was 3 years old. Table 1 presents the number and percentage of items selected from the collected data. As we excluded cases with one or both unanswered questionnaires on pool use frequency at T1 and T2 (*n* = 20,599), data from 79,687 mother-child pairs were analyzed (Fig. 1). The questionnaire at T1 included the following question: “Frequency of playing in a swimming pool (not a pool at home) after 12 months of age,” which used a five-point response scale (1 = seldom, 2 = once per month, 3 = 2–3 times per month, 4 = once per week, and 5 = ≥ 2 times per week). The questionnaire at T2 included the following question: “Frequency of going to a swimming pool after 2 years of age,” which used a six-point response scale (1 = seldom, 2 = a few times only in the summer, 3 = once per month, 4 = 2–3 times per month, 5 = once per week, and 6 = ≥ 2 times per week). In the JECS questionnaire, only the T2 questionnaire included the response option “a few times only in the summer.” However, because this was not considered regular swimming, it was treated as equivalent to “seldom.” Therefore, in this study, T2 responses were evaluated on a five-point scale for regular swimming use, similar to the T1 responses.

**Table 1:** Number and percentage of pool use frequency at T1 and T2

	T1^a^	T2^b^
Five or six selection items^c^	n	n
	(%)	(%)
Never or almost never	61,782	32,187
	(77.5)	(40.4)
A few times only in the summer	-	37,862
		(47.5)
Once per month	4,620	1,192
	(5.8)	(1.5)
2–3 times per month	5,034	1,472
	(6.3)	(1.9)
Once per week	3,064	2,637
	(3.9)	(3.3)
≥ 2 times per week	5,187	4,337
	(6.5)	(5.4)
Categorized into two selection items^d^	n	n
	(%)	(%)
Seldom	61,782	70,049
	(77.5)	(87.9)
Once a month or more	17,905	9,638
	(22.5)	(12.1)

^a^T1 refers to the questionnaire conducted at the age of 1.5 years. It asks about the frequency of pool use from age 1 to 1.5 years

^b^T2 refers to the questionnaire conducted at the age of 3 years. It asks about the frequency of pool use from age 2 to 3 years

^c^1.5-year-olds whose data were received for the five selection items except “a few times only in the summer”

^d^1.5-year-olds categorized by the four choices other than “seldom” into “once a month or more.” For 3-year-olds, “seldom” and “a few times only in the summer” were grouped as “seldom.” The other four selection items were categorized as “once a month or more”

This study focused on identifying whether children had developed a regular swimming habit, and based on this, categorized the pattern of continued swimming pool use into four frequency categories as follows: The frequency of children’s pool use from age 1 to 1.5 years was classified into two groups based on responses obtained in the questionnaire at T1: group a (*n* = 61,782; 77.5%) if the response was “1” (seldom) and group b (*n* = 17,905; 22.5%) if any of the other four scale items were selected (indicating regular use, once a month or more). The frequency of children’s pool use from age 2 to 3 years was classified into two groups based on responses obtained in the questionnaire at T2: group c (*n* = 70,049; 87.9%) if the response was “1” or “2” and group d (*n* = 9,638; 12.1%) if any of the other four scale items were selected (indicating regular use, once a month or more). Groups a and b, reflecting the frequency of pool use from ages 1 to 1.5 years, and groups c and d, reflecting the frequency of pool use from ages 2 to 3 years, were combined and redefined into four continuation patterns: Group A (groups a and c) as “seldom used the swimming pool during the ages of 1–3 years;” Group B (groups a and d) as “seldom used the swimming pool during the ages of 1–1.5 years, but started using it once a month or more from 2 to 3 years of age”; Group C (groups b and c) as “used the swimming pool once a month or more during 1–1.5 years of age, but stopped using it from 2 to 3 years of age;” and Group D (groups b and d) as “continued to use the swimming pool once a month or more from 1 to 3 years of age.”

### Outcomes

Neurodevelopment in children was measured using a developmental evaluation tool, the Ages and Stages Questionnaire, Third Edition (ASQ-3) [18], a developmental evaluation tool completed by parents or guardians to evaluate children aged 1–66 months. The ASQ-3 assesses five areas: communication, gross motor skills, fine motor skills, problem-solving, and personal-social skills. For this study, the Japanese version of the ASQ-3 (J-ASQ-3) was utilized [19]. Gross motor skills and fine motor skills, assessed using the J-ASQ-3, served as the primary indices of motor developmental outcomes. Evaluations were performed at targeted ages: 1.5, 2, 2.5, and 3 years of age. Cutoff scores recommended by the original ASQ-3 [18] were used to dichotomize outcomes: 37.38 at 1.5 years, 38.07 at 2 years, 36.14 at 2.5 years, and 36.99 at 3 years for gross motor function; and 34.32 at 1.5 years, 35.16 at 2 years, 19.25 at 2.5 years, and 18.07 at 3 years for fine motor function. Scores below these cutoffs indicated a need for specialist evaluation [18]. The original ASQ-3 cutoffs were chosen over Japanese-specific cutoffs (calculated from data collected in limited geographical locations in Japan) [20], to ensure broader applicability and generalizability of findings.

### Covariates

The following covariates were included in the logistic regression analysis: highest maternal educational level (junior high school or high school/technical junior college, technical/vocational college or associate degree/Bachelor’s degree, and postgraduate degree), annual household income in Japanese yen (< 2; 2–<4; 4–<6; 6–<8; 8–<10; ≥10 million), sex of the child (male/female), physical anomalies (no/yes), child body mass index, frequency of mother playing with the child (seldom or 1–3 times per month, 1–2 times per week, 3–4 times per week, or at least 5 times per week), frequency of taking the child out of the house (somewhere other than the childcare facility; seldom or 1–3 times per month, 1–2 times per week, 3–4 times per week, or at least 5 times per week), and childcare facility or preschool attendance (daycare center or nursery; yes/no).

### Statistical analysis

#### Maternal and child characteristics

Below, we first present basic statistics on covariates and outcomes in this study, both overall and by group.

### Logistic regression analysis

Missing data were handled using multiple imputations based on the assumption that the data were missing at random. This approach was employed to enhance the robustness of the analysis results and mitigate potential bias arising from incomplete responses or selection. Odds ratios (ORs) and 95% confidence intervals (95% CIs) for the outcome variables were estimated using 20 appropriately imputed models.

After performing multiple imputation, logistic regression analysis was conducted to estimate the associations between different levels of swimming pool use frequency at T1 and T2 and delays in gross and fine motor function development (0 = pass, 1 = fail) assessed by the J-ASQ-3. Adjusted ORs (aORs) and 95% CIs for gross and fine motor function, using “seldom” as the reference category, across levels of swimming pool use frequency: “once per month,” “2–3 times per month,” “once per week,” and “≥2 times per week.” Similarly, using the same analytical approach, we evaluated the associations between patterns of swimming pool use frequency at T1 and T2 and the development of delays in gross and fine motor function (0 = pass, 1 = fail) in the J-ASQ-3. We calculated aORs and 95% CIs for gross and fine motor function as outcome variables, using Group A (children who seldom used swimming pools during ages 1–3 years) as the reference category, for Groups B, C, and D. All analyses were performed using SPSS version 28 (IBM Corp., Tokyo, Japan). The statistical significance level was set as 5% for all analyses.

## Results

### Maternal and child characteristics

Table 2 presents the maternal and child characteristics, which serves as covariates in each group. Table 3 outlines the number and percentage of children in each group, along with results of chi-square tests for gross and fine motor function development. Overall, 79,687 valid respondents completed questionnaires for both 1.5- and 3-year-old children. Patterns of continued swimming pool use were categorized by frequency as follows: 69.8% (*n* = 55,625) were in Group A, 7.7% (*n* = 6,157) were in Group B, 18.1% (*n* = 14,424) were in Group C, and 4.4% (*n* = 3,481) were in Group D. Chi-square test results indicated a significant association between the four groups of exposure factors and ASQ-3 (gross and fine motor function) cutoff scores across all included age groups (*p* < 0.05).

**Table 2:** Maternal and child characteristics

Variables			Continuation pattern of the frequency of using the swimming pool			
		Total	Group Aª	Group B^b^	Group C^c^	Group D^d^
		n = 79,687	n = 55,625	n = 6,157	n = 14,424	n = 3,481
Children		n	n	n	n	n
Sex		(%)	(%)	(%)	(%)	(%)
Male		40,806	28,429	3,114	7,386	1,877
		(51.2)	(51.1)	(50.6)	(51.2)	(53.9)
Female		38,881	27,196	3,043	7,038	1,604
		(48.8)	(48.9)	(49.4)	(48.8)	(46.1)
Physical anomalies		n	n	n	n	n
		(%)	(%)	(%)	(%)	(%)
No		67,108	46,887	5,201	12,087	2,933
		(84.2)	(84.3)	(84.5)	(83.8)	(84.3)
Yes		7,703	5,263	605	1,498	337
		(9.7)	(9.5)	(9.8)	(10.4)	(9.7)
Missing		4876	3475	351	839	211
		(6.1)	(6.2)	(5.7)	(5.8)	(6.1)
Body mass index (kg/m^2^)		mean	mean	mean	mean	mean
		(SD)	(SD)	(SD)	(SD)	(SD)
1.5 years		16.6	16.6	16.7	16.6	16.7
		(1.5)	(1.5)	(1.5)	(1.5)	(1.5)
Missing		9,400	6,610	725	1,675	390
2 years		16.4	16.3	16.4	16.5	16.5
		(1.3)	(1.4)	(1.4)	(1.3)	(1.3)
Missing		7,743	5,343	566	1,476	358
2.5 years		16.2	16.2	16.3	16.2	16.3
		(1.3)	(1.3)	(1.3)	(1.3)	(1.3)
Missing		9,637	6,799	709	1,742	387
3 years		16.0	16.0	16.0	16.1	16.1
		(1.3)	(1.3)	(1.2)	(1.3)	(1.3)
Missing		6,318	4,520	422	1,146	230
Mothers		n	n	n	n	n
Annual household income (million Japanese yen)		(%)	(%)	(%)	(%)	(%)
< 2		3,912	2,661	310	753	188
		(4.9)	(4.8)	(5.0)	(5.2)	(5.4)
2– < 4		23,709	16,457	1,864	4,387	1,001
		(29.8)	(29.6)	(30.3)	(30.4)	(28.8)
4– < 6		23,685	16,482	1,832	4,315	1,056
		(29.7)	(29.6)	(29.8)	(29.9)	(30.3)
6– < 8		11,593	8,172	882	2,027	512
		(14.5)	(14.7)	(14.3)	(14.1)	(14.7)
8– < 10		4,785	3,389	391	812	193
		(6.0)	(6.1)	(6.4)	(5.6)	(5.5)
≥ 10		3,015	2,129	216	541	129
		(3.8)	(3.8)	(3.5)	(3.8)	(3.7)
Missing		8,988	6,335	662	1,589	402
		(11.3)	(11.4)	(10.8)	(11.0)	(11.5)
Maternal highest level of education		n	n	n	n	n
		(%)	(%)	(%)	(%)	(%)
Junior high school or high school		26,481	18,750	1,951	4,841	939
		(33.2)	(33.7)	(31.7)	(33.6)	(27.0)
Technical junior college, technical/vocational college, or Associate degree		34,008	23,719	2,591	6,236	1,462
		(42.7)	(42.6)	(42.1)	(43.2)	(42.0)
Bachelor degree or Postgraduate degree		18,369	12,596	1,537	3,204	1,032
		(23.1)	(22.6)	(25.0)	(22.2)	(29.6)
Missing		829	560	78	143	48
		(1.0)	(1.0)	(1.3)	(1.0)	(1.4)
Lifestyle factors		n	n	n	n	n
Maternal frequency of playing with the child		(%)	(%)	(%)	(%)	(%)
1 year	Seldom or 1–3 times per month	487	322	32	104	29
		(0.6)	(0.6)	(0.5)	(0.7)	(0.8)
	1–2 times per week	3999	2573	322	885	219
		(5.0)	(4.6)	(5.2)	(6.1)	(6.3)
	3–4 times per week	3831	2464	296	876	195
		(4.8)	(4.4)	(4.8)	(6.1)	(5.6)
	≥ 5 times per week	70,149	49,467	5418	12,290	2974
		(88.0)	(88.9)	(88.0)	(85.2)	(85.4)
	Missing	1,221	799	89	269	64
		(1.5)	(1.4)	(1.4)	(1.9)	(1.8)
2 years	Seldom or 1–3 times per month	1,108	725	85	249	49
		(1.4)	(1.3)	(1.4)	(1.7)	(1.4)
	1–2 times per week	13,172	8,352	1,132	3,048	640
		(16.5)	(15.0)	(18.4)	(21.1)	(18.4)
	3–4 times per week	8,565	5,654	701	1,815	395
		(10.7)	(10.2)	(11.4)	(12.6)	(11.3)
	≥ 5 times per week	55,157	39,803	4,096	8,964	2,294
		(69.2)	(71.6)	(66.5)	(62.1)	(65.9)
	Missing	1,685	1,091	143	348	103
		(2.1)	(2.0)	(2.3)	(2.4)	(3.0)
3 years	Seldom or 1–3 times per month	1,940	1,292	150	412	86
		(2.4)	(2.3)	(2.4)	(2.9)	(2.5)
	1–2 times per week	16,560	10,691	1,483	3,615	771
		(20.8)	(19.2)	(24.1)	(25.1)	(22.1)
	3–4 times per week	10,680	7,114	923	2,156	487
		(13.4)	(12.8)	(15.0)	(14.9)	(14.0)
	≥ 5 times per week	50,201	36,312	3,572	8,195	2,122
		(63.0)	(65.3)	(58.0)	(56.8)	(61.0)
	Missing	306	216	29	46	15
		(0.4)	(0.4)	(0.5)	(0.3)	(0.4)
Frequency of taking the child out of the house (to somewhere other than the childcare facility)		n (%)	n (%)	n (%)	n (%)	n (%)
1 year	Seldom or 1–3 times per month	2,979	2,050	221	587	121
		(3.7)	(3.7)	(3.6)	(4.1)	(3.5)
	1–2 times per week	21,739	14,776	1,648	4,435	880
		(27.3)	(26.6)	(26.8)	(30.7)	(25.3)
	3–4 times per week	21,938	15,976	1,601	3,549	812
		(27.5)	(28.7)	(26.0)	(24.6)	(23.3)
	≥ 5 times per week	31,886	22,075	2,604	5,598	1,609
		(40.0)	(39.7)	(42.3)	(38.8)	(46.2)
	Missing	1,145	748	83	255	59
		(1.4)	(1.3)	(1.3)	(1.8)	(1.7)
2 years	Seldom or 1–3 times per month	3,540	2,403	265	743	129
		(4.4)	(4.3)	(4.3)	(5.2)	(3.7)
	1–2 times per week	32,687	21,508	2,701	7,013	1,465
		(41.0)	(38.7)	(43.9)	(48.6)	(42.1)
	3–4 times per week	17,558	12,878	1,246	2,823	611
		(22.0)	(23.2)	(20.2)	(19.6)	(17.6)
	≥ 5 times per week	24,256	17,775	1,802	3,500	1,179
		(30.4)	(32.0)	(29.3)	(24.3)	(33.9)
	Missing	1,646	1,061	143	345	97
		(2.1)	(1.9)	(2.3)	(2.4)	(2.8)
3 years	Seldom or 1–3 times per month	4,699	3,147	400	963	189
		(5.9)	(5.7)	(6.5)	(6.7)	(5.4)
	1–2 times per week	36,838	24,302	3,179	7,655	1,702
		(46.2)	(43.7)	(51.6)	(53.1)	(48.9)
	3–4 times per week	17,409	12,840	1,165	2,782	622
		(21.8)	(23.1)	(18.9)	(19.3)	(17.9)
	≥ 5 times per week	20,546	15,202	1,392	2,989	963
		(25.8)	(27.3)	(22.6)	(20.7)	(27.7)
	Missing	195	134	21	35	5
		(0.2)	(0.2)	(0.3)	(0.2)	(0.1)
Attendance at a childcare facility / a preschool (daycare center or nursery)		n (%)	n (%)	n (%)	n (%)	n (%)
1 year	Yes	20,224	12,037	1,768	5,268	1,151
		(25.4)	(21.6)	(28.7)	(36.5)	(33.1)
	No	58,247	42,816	4,290	8,875	2,266
		(73.1)	(77.0)	(69.7)	(61.5)	(65.1)
	Missing	1,216	772	99	281	64
		(1.5)	(1.4)	(1.6)	(1.9)	(1.8)
2 years	Yes	38,080	23,444	3,508	9,148	1,980
		(47.8)	(42.1)	(57.0)	(63.4)	(56.9)
	No	39,616	30,896	2,488	4,845	1,387
		(49.7)	(55.5)	(40.4)	(33.6)	(39.8)
	Missing	1,991	1,285	161	431	114
		(2.5)	(2.3)	(2.6)	(3.0)	(3.3)
3 years	Yes	48,499	31,018	4,567	10,409	2,505
		(60.9)	(55.8)	(74.2)	(72.2)	(72.0)
	No	28,772	23,094	1,368	3,459	851
		(36.1)	(41.5)	(22.2)	(24.0)	(24.4)
	Missing	2,416	1,513	222	556	125
		(3.0)	(2.7)	(3.6)	(3.9)	(3.6)

*SD* Standard deviation

^a^Group A, children who seldom go to a swimming pool during 1 to 3 years of age.

^b^Group B, children who seldom used a swimming pool during 1–1.5 years of age but started swimming once a month or more from 2 to 3 years of age

^c^Group C, children who went to a swimming pool once a month or more during 1–1.5 years of age but quit swimming from 2 to 3 years of age

^d^Group D, children who continued to use the swimming pool once a month or more from 1 to 3 years of age

**Table 3:** Characteristics of outcomes

			Continuation pattern of the frequency of using the swimming pool				
Variables		Total	Group Aª	Group B^b^	Group C^c^	Group D^d^	
		*n* = 79,687	*n* = 55,625	*n* = 6,157	*n* = 14,424	*n* = 3,481	p^e^
Gross motor		n	n	n	n	n	
		(%)	(%)	(%)	(%)	(%)	
1.5 years	Pass^f^	65,875	46,171	5,083	11,769	2,852	*p* < 0.001
		(82.7)	(83.0)	(82.6)	(81.6)	(81.9)	
	Fail^g^	3,380	2,560	266	445	109	
		(4.2)	(4.6)	(4.3)	(3.1)	(3.1)	
	Missing	10,432	6,894	808	2,210	520	
		(13.1)	(12.4)	(13.1)	(15.3)	(14.9)	
2 years	Pass	68,542	47,867	5,285	12,427	2,963	*p* < 0.001
		(86.0)	(86.1)	(85.8)	(86.2)	(85.1)	
	Fail	4,602	3,460	364	629	149	
		(5.8)	(6.2)	(5.9)	(4.4)	(4.3)	
	Missing	6,543	4,298	508	1,368	369	
		(8.2)	(7.7)	(8.3)	(9.5)	(10.6)	
2.5 years	Pass	69,097	48,261	5,353	12,477	3,006	*p* < 0.001
		(86.7)	(86.8)	(86.9)	(86.5)	(86.4)	
	Fail	3,406	2,619	258	426	103	
		(4.3)	(4.7)	(4.2)	(3.0)	(3.0)	
	Missing	7,184	4,745	546	1,521	372	
		(9.0)	(8.5)	(8.9)	(10.5)	(10.7)	
3 years	Pass	73,242	51,053	5,647	13,325	3,217	*p* < 0.001
		(91.9)	(91.8)	(91.7)	(92.4)	(92.4)	
	Fail	3,648	2,777	268	495	108	
		(4.6)	(5.0)	(4.4)	(3.4)	(3.1)	
	Missing	2,797	1,795	242	604	156	
		(3.5)	(3.2)	(3.9)	(4.2)	(4.5)	
Fine motor		n	n	n	n	n	
		(%)	(%)	(%)	(%)	(%)	
1.5 years	Pass	63,557	44,415	4,938	11,408	2,796	*p* < 0.001
		(79.8)	(79.8)	(80.2)	(79.1)	(80.3)	
	Fail	5,663	4,295	406	797	165	
		(7.1)	(7.7)	(6.6)	(5.5)	(4.7)	
	Missing	10,467	6,915	813	2,219	520	
		(13.1)	(12.4)	(13.2)	(15.4)	(14.9)	
2 years	Pass	68,959	48,135	5,371	12,490	2,963	*p* < 0.001
		(86.5)	(86.5)	(87.2)	(86.6)	(85.1)	
	Fail	4,116	3,150	272	549	145	
		(5.2)	(5.7)	(4.4)	(3.8)	(4.2)	
	Missing	6,612	4,340	514	1,385	373	
		(8.3)	(7.8)	(8.3)	(9.6)	(10.7)	
2.5 years	Pass	70,349	49,273	5,440	12,583	3,053	*p* < 0.001
		(88.3)	(88.6)	(88.4)	(87.2)	(87.7)	
	Fail	1,848	1,394	147	260	47	
		(2.3)	(2.5)	(2.4)	(1.8)	(1.4)	
	Missing	7,490	4,958	570	1,581	381	
		(9.4)	(8.9)	(9.3)	(11.0)	(10.9)	
3 years	Pass	73,970	51,628	5,692	13,425	3,225	*p* < 0.001
		(92.8)	(92.8)	(92.4)	(93.1)	(92.6)	
	Fail	2,633	1,998	198	362	75	
		(3.3)	(3.6)	(3.2)	(2.5)	(2.2)	
	Missing	3,084	1,999	267	637	181	
		(3.9)	(3.6)	(4.3)	(4.4)	(5.2)	

^a^Group A, children who seldom went to a swimming pool during 1–3 years of age

^b^Group B, children who seldom went to a swimming pool during 1–1.5 years of age, but started swimming once a month or more from 2 to 3 years of age

^c^Group C, children who went to a swimming pool once a month or more during 1–1.5 years of age but quit swimming from 2 to 3 years of age.

^d^Group D, children continued to use the swimming pool once a month or more from 1 to 3 years of age

^e^The chi-squared test for proportion was used to assess the association between the exposures and outcomes

^f^Above cutoff value

^g^Below cutoff value

### Associations of levels of pool use frequency with the development of gross and fine motor function in children

We examined whether gross and fine motor function development differed based on various levels of swimming pool use frequency at T1 and T2 (Tables 4 and 5). At T1, a significant negative association was observed between delays in gross motor function development and all pool use frequency levels across all ages, except for the “once per month” frequency at age 2 years. Similarly, delays in fine motor function development showed significantly negative associations with all levels of pool use frequency at ages 1.5 years, 2.5 years and 3 years. Additionally, significant negative associations were observed for “once per month” and “≥2 times per week” at ages 2 years. When “seldom” was used as the reference, no substantial differences were observed in the ORs across the levels of pool use frequency from “once per month” to “≥2 times per week”; similar ORs were confirmed. At T2, significant negative associations were observed between delays in gross motor function development and the “once per week” frequency at age 2 years and “once per month” frequency at age 2.5 years. Regarding delays in fine motor function development, significant negative associations were observed with the “once per month” and “2–3 times per month” frequencies at 2.5 years and 3 years, respectively, as well as with the “once per week” frequency at 3 years.

**Table 4:** Associations between each level of the frequency of pool use and the development of gross motor function

Time	Levels of swimming pool use frequency	1.5 years				2 years				2.5 years				3 years			
		aOR^c^	95% CI^d^			aOR	95% CI			aOR	95% CI			aOR	95% CI		
			lower		upper		lower		upper		lower		upper		lower		upper
T1^a^	Seldom (reference)	―	―		―	―	―		―	―	―		―	―	―		―
	Once per month	**0.59**	**(0.52**	**-**	**0.68)**	0.88	(0.78	-	1.00)	**0.70**	**(0.61**	**-**	**0.80)**	**0.78**	**(0.68**	**-**	**0.89)**
	2–3 times per month	**0.70**	**(0.60**	**-**	**0.81)**	**0.88**	**(0.78**	**-**	**0.99)**	**0.75**	**(0.66**	**-**	**0.87)**	**0.83**	**(0.72**	**-**	**0.95)**
	Once per week	**0.70**	**(0.57**	**-**	**0.85)**	**0.79**	**(0.67**	**-**	**0.93)**	**0.72**	**(0.59**	**-**	**0.87)**	**0.78**	**(0.64**	**-**	**0.95)**
	≥ 2 times per week	**0.72**	**(0.62**	**-**	**0.84)**	**0.71**	**(0.62**	**-**	**0.81)**	**0.56**	**(0.46**	**-**	**0.67)**	**0.70**	**(0.60**	**-**	**0.82)**
T2^b^	Seldom (reference)	―	―		―	―	―		―	―	―		―	―	―		―
	Once per month	―	―		―	1.02	(0.87	-	1.21)	**0.82**	**(0.69**	**-**	**0.97)**	0.90	(0.76	-	1.07)
	2–3 times per month	―	―		―	1.10	(0.94	-	1.28)	0.83	(0.66	-	1.04)	0.93	(0.76	-	1.12)
	Once per week	―	―		―	**0.84**	**(0.70**	**-**	**1.00)**	0.82	(0.66	-	1.01)	0.86	(0.71	-	1.05)
	≥ 2 times per week	―	―		―	0.93	(0.81	-	1.07)	0.96	(0.82	-	1.13)	0.88	(0.75	-	1.03)

^a^T1 refers to the questionnaire conducted at age 1.5 years, asking about the frequency of pool use from age 1 to 1.5 years

^b^T2 refers to the questionnaire conducted at the age of 3 years, asking about the frequency of pool use from age 2 to 3 years

^c^aOR, adjusted odds ratio; ^d^95% CI, 95% confidence intervals

Bold values indicate statistically significant results (*p* < 0.05)

**Table 5:** Associations between each level of the frequency of pool use and the development of fine motor function

Time	Levels of swimming pool use frequency	1.5 years				2 years				2.5 years				3 years			
		aOR^c^	95% CI^d^			aOR	95% CI			aOR	95% CI			aOR	95% CI		
			lower		upper		lower		upper		lower		upper		lower		upper
T1^a^	Seldom (reference)	―	―		―	―	―		―	―	―		―	―	―		―
	Once per month	**0.72**	**(0.65**	-	**0.81)**	**0.79**	**(0.70**	-	**0.91)**	**0.53**	**(0.43**	-	**0.66)**	**0.64**	**(0.55**	-	**0.75)**
	2–3 times per month	**0.78**	**(0.70**	-	**0.87)**	0.88	(0.78	**-**	1.00)	**0.66**	**(0.54**	-	**0.80)**	**0.63**	**(0.52**	-	**0.75)**
	Once per week	**0.68**	**(0.58**	-	**0.79)**	0.86	(0.72	**-**	1.02)	**0.69**	**(0.53**	-	**0.90)**	**0.67**	**(0.53**	-	**0.85)**
	≥ 2 times per week	**0.72**	**(0.64**	-	**0.82)**	**0.65**	**(0.55**	-	**0.76)**	**0.63**	**(0.50**	-	**0.80)**	**0.72**	**(0.60**	-	**0.87)**
T2^b^	Seldom (reference)	―	―		―	―	―		―	―	―		―	―	―		―
	Once per month	―	―		―	0.86	(0.72	**-**	1.04)	**0.55**	**(0.40**	-	**0.75)**	**0.54**	**(0.39**	-	**0.74)**
	2–3 times per month	―	―		―	0.89	(0.72	**-**	1.10)	**0.66**	**(0.47**	-	**0.92)**	**0.69**	**(0.52**	-	**0.91)**
	Once per week	―	―		―	0.83	(0.69	**-**	1.00)	0.87	(0.66	**-**	1.15)	**0.77**	**(0.60**	-	**0.99)**
	≥ 2 times per week	―	―		―	0.92	(0.79	**-**	1.07)	0.96	(0.77	**-**	1.20)	0.95	(0.79	**-**	1.15)

^a^T1 refers to the questionnaire conducted at age 1.5 years, asking about the frequency of pool use from age 1 to 1.5 years.

^b^T2 refers to the questionnaire conducted at the age of 3 years, asking about the frequency of pool use from age 2 to 3 years

^c^aOR, adjusted odds ratio; ^d^95% CI, 95% confidence intervals

Bold values indicate statistically significant results (*p* < 0.05)

### Association of the pattern of continued swimming pool use with gross motor function

**Fig. 2:**
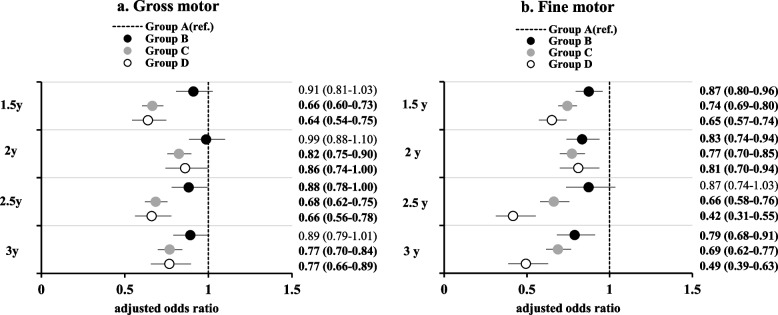
Relationship between the level of continued pool use frequency and gross and fine motor development. Group A: Children who seldom used the swimming pool during 1–3 years of age, used as a reference. Group B: Children who seldom used the swimming pool during 1–1.5 years of age but started swimming once a month or more from 2 to 3 years of age. Group C: Children who used the swimming pool once a month or more during 1 to 1.5 years of age but quit swimming by 2–3 years of age Group D: Children who continued to use the swimming pool once a month or more from 1 to 3 years of age. 1.5y, 1.5 years (18 months); 2y, 2 years (24 months); 2.5y, 2.5 years (30 months); 3y, 3 years (36 months); aOR, adjusted odds ratio; 95% CI, 95% confidence intervals. Bold values indicate statistically significant results (*p* < 0.05)

In terms of gross motor function, significant negative associations were observed at only age 2.5 years for Group B (2.5y, aOR: 0.88, 95% CI: 0.78–1.00) (Fig. 2a). In Group C, a significantly negative association with gross motor function development delay was observed across all ages (1.5y, aOR: 0.66, 95% CI: 0.60–0.73; 2y, aOR: 0.82, 95% CI: 0.75–0.90; 2.5y, aOR: 0.68, 95% CI: 0.62–0.75; 3y, aOR: 0.77, 95% CI: 0.70–0.84). This indicates that the positive effect of swimming on gross motor function development persisted until 3 years of age, even if swimming was started at 1 year of age and stopped at 2 years of age. In Group D, a significantly negative association with gross motor function development delay was observed across all ages (1.5y, aOR: 0.64, 95% CI: 0.54–0.75; 2y, aOR: 0.86, 95% CI: 0.74–1.00; 2.5y, aOR: 0.66, 95% CI: 0.56–0.78; 3y, aOR: 0.77, 95% CI: 0.66–0.89). This indicates that the positive effect of starting swimming at 1 year of age on gross motor function development persisted until 3 years of age.

### Association of the pattern of continued swimming pool use with fine motor function

In Group B, a significantly negative association with delayed fine motor function was observed at 1.5 years of age (aOR: 0.87, 95% CI: 0.80–0.96), 2 years of age (aOR: 0.83, 95% CI: 0.74–0.94) and 3 years of age (aOR: 0.79, 95% CI: 0.68–0.91) (Fig. 2b). In Group C, significant negative associations with delayed fine motor function were observed at all ages (1.5y, aOR: 0.74, 95% CI: 0.69–0.80; 2y, aOR: 0.77, 95% CI: 0.70–0.85; 2.5y, aOR: 0.66, 95% CI: 0.58–0.76; 3y, aOR: 0.69, 95% CI: 0.62–0.77). This indicates that the positive effects on fine motor function development continued until 3 years of age, even when swimming was started at 1 year of age and stopped at 2 years of age. Similarly, in Group D, significant negative associations were also observed at all ages (1.5 years, aOR: 0.65, 95% CI: 0.57–0.74; 2 years, aOR: 0.81, 95% CI: 0.70–0.94; 2.5 years, aOR: 0.42, 95% CI: 0.31–0.55; and 3 years, aOR: 0.49, 95% CI: 0.39–0.63). This indicates that the effect of starting swimming from 1 year of age on fine motor function development persisted until 3 years of age. Interestingly, this effect was more pronounced after 2.5 years of age.

## Discussion

This study found that initiating swimming at approximately 1 year of age positively influenced the development of both gross and fine motor function up to the age of 3 years. Specifically, the impact on gross motor function development was observed earlier than that on fine motor function development. Initiating swimming approximately 1 year of age resulted in sustained positive effects on gross motor development throughout the study period, regardless of whether children ceased swimming at 2 years of age or continued until 3 years of age. Regarding fine motor function development, initiating swimming at approximately 1 year of age initially showed no significant difference in continuation up to 2 years of age. However, starting swimming at approximately 1 year of age led to a notable improvement in fine motor development that became apparent after the age of 2.5 years.

In children who started swimming from 1 to 3 years of age, a positive motor development effect was observed in those who started swimming from 1 year of age, albeit almost no effect was observed in those who started swimming after 2 years of age. These results suggest that swimming experience of children aged up to 2 years may influence gross and fine motor function development. Gallahue and Ozmun reported motor developmental stages from fetal life through childhood, delineating four stages in their word titled “*The phases and stages of motor development* [21].” They categorized the period from birth to 2 years as the “rudimentary movement phase” and from 2 to 7 years as the “fundamental movement phase.” During the rudimentary movement phase, they emphasized the critical motor development milestones up to 2 years of age, including acquiring motor skills such as maintaining upright posture maintenance, independent walking, and grasping and manipulation [22, 23]. This phase is crucial for the development of both gross and fine motor function develop [24].

The nervous system of children undergoes significant development from birth through early childhood [25]. By the age of 3 years, the number of nerve cells in the brain reaches a level comparable to that in adults, forming essential neural circuits [26]. Motor competence in infancy is characterized by achieving gross motor milestones, but substantial individual differences in motor development are well-documented [27]. Around 1 year of age, infants typically transition from crawling on their stomach to crawling on hands and knees, standing with support, and walking independently. These changes in posture mark a critical stage in locomotion development [28–30]. Such postural changes are pivotal for the development of motor skills including core body function, balance, muscle strength, and visual function, all of which are crucial during physical activity. Moreover, early movement experiences, start approximately at 1 year of age, play a vital role in shaping the nervous system [31]. Continuous baby swimming experiences during this developmental period involve movements such as floating, swimming, and diving, which are unique to aquatic environments. These experiences provide broad kinesthetic stimuli that contribute to the development of various motor skills and abilities.

The lack of observed effects on gross motor function development at age 3 years in cases where swimming was initiated at approximately age 2 years may be attributed to the duration of swimming experience. Immediate effects on motor development are unlikely because it takes time for children to adjust to water activities. Since this study followed children only up to age 3 years, it could not assess whether starting swimming at age 2 years would continue to impact motor development beyond age 3 years. Further research is needed to explore this aspect comprehensively.

Numerous studies on baby swimming have documented varied respiratory health effects, both positive and negative [1, 32–34], although consistent evidence remains elusive. Previous research has consistently shown that baby swimming positively influences motor competence [11–14], a finding corroborated by the present study. Although earlier studies cited small sample sizes and on-site interventions as limitations, this study utilized a large cohort and relied solely on questionnaires without direct intervention. Despite the methodological differences, both the present and previous studies concur that swimming experiences before the age of 3 years contribute significantly to motor competence.

This study had several limitations. First, it lacked information on how children and their parents used the swimming pool, as the questionnaire did not assess specific details such as attendance at swimming schools or whether children used pool facilities with their parents. Second, being a large-scale cohort survey relying solely on questionnaires, the study did not gather information on the content of the swimming programs, precluding determination of the most effective program for motor competence. Third, there was a gap in data regarding the frequency of swimming pool use for children aged 1.5–2 years, as the questionnaire covered only covered time points at 1.5 and 3 years. The 1.5-year questionnaire queried the frequency of swimming pool use from age 1 to approximately 1.5 years, whereas the 3-year questionnaire usage from age 2 to approximately 3 years. Consequently, data for the 1.5–2-year age range was not captured. Furthermore, the questionnaire did not accurately capture seasonal variations in swimming. Finally, although gross and fine motor function development can be influenced by activities beyond swimming, the JECS questionnaire used in this study did not assess other aspects of physical activity, preventing adjustment for these factors as covariates.

Despite its limitations, the study also had strengths. Firstly, it utilized a large-scale prospective cohort. Additionally, it evaluated the development of gross and fine motor function using the standardized J-ASQ-3 based on the data obtained. Furthermore, by adjusting for numerous potential confounders, the study was able to elucidate the impact of swimming experience from age 1 year on the development of motor functions up to age 3 years. In the future, similar studies will need to be performed with children > 3 years to further clarify the effects of swimming experiences on motor competence in infancy and early childhood using longitudinal, large-scale epidemiological studies.

## Conclusion

In conclusion, our results suggest that swimming experience starting from around age 1 year is positively associated with gross and fine motor function development. Starting swimming at approximately 1 year of age consistently sustained gross motor development effects until the age of 3 years, irrespective of whether the children quit swimming at the age of 2 years or continued swimming until 3 years of age. Regarding fine motor function, our findings indicate that the positive effects of starting swimming at approximately 1 year of age become noticeable after 2.5 years of age. The findings from this study make a significant contribution to the field of motor development in children, particularly in the context of baby swimming. Studies using large-scale prospectively collected cohorts like this one have been relatively rare. Moving forward, it is essential to conduct detailed investigations into interventions and aquatic exercise programs that can enhance motor development from infancy through early childhood. This research will help further our understanding and optimize practices aimed at improving children’s motor skills through swimming and similar activities.

## Data Availability

Data are unsuitable for public deposition because of ethical restrictions and the legal framework of Japan. The Act on the Protection of Personal Information (Act No. 57 of May 30, 2003, amended on September 9, 2015) prohibits the public from depositing data containing personal information. The Ethical Guidelines for Medical and Health Research Involving Human Subjects enforced by the Japan Ministry of Education, Culture, Sports, Science and Technology and the Ministry of Health, Labour, and Welfare also restrict the open sharing of epidemiological data. All inquiries regarding data access should be sent to jecs-en@nies.go.jp. The person responsible for handling the inquiries sent to this email address is Dr. Shoji F. Nakayama, JECS Program Office, National Institute for Environmental Studies.

## Abbreviations

aOR: adjusted odds ratio
ASQ-3: the Ages and Stages Questionnaire, Third Edition
CI: Confidence interval
JECS: the Japan Environment and Children’s Study

## References

[1] Irahara M, Yamamoto-Hanada K, Yang L, Saito-Abe M, Sato M, Inuzuka Y, et al. Impact of swimming school attendance in 3-year-old children with wheeze and rhinitis at age 5 years: a prospective birth cohort study in Tokyo. PLoS ONE. 2020;15:1–12. 10.1371/journal.pone.0234161 PMC7282662 32516323

[2] Martins M, Costa A, Costa MJ, Marinho DA, Barbosa TM. Correction: interactional response during infants’ aquatic sessions. Sport Med Int Open. 2020;4:E70-5. 10.1055/a-1201-4522 PMC7413845 32782924

[3] Hayashi Y. Baby swimming no rekishi [History of baby swimming]. Japanese J Sci Swim Water Exerc. 1998;31:32–40.

[4] Kouyama R. Baby swimming. Japanese J Phys Fit Sport Med. 2006;55:100–1.

[5] Meguro S. Baby swimming shidou riron [Baby swimming instruction theory]. Tokyo: Kankyou Kougakusha; 2016.

[6] Erbaugh SJ. Effects of aquatic training on swimming skill development of preschool children. Percept Mot Skills. 1986;62:439–46. 3503250 10.2466/pms.1986.62.2.439

[7] Zelazo PR, Weiss MJ. Infant swimming behaviors: cognitive control and the influence of experience. J Cogn Dev. 2006;7:1–25.

[8] Benčuriková L. Dynamic balance in water and its influence on children’s swimming ability. Int Q Sport Sci. 2009;9:29–37.

[9] Rocha HA, Marinho DA, Jidovtseff B, Silva AJ, Costa AM. Influence of regular soccer or swimming practice on gross motor development in childhood. Motricidade. 2016;12:33–43.

[10] Asher D, Roth D, Frumer-Hadar M. The effect of structured water activity on motor ability, parental attitude, self-concept, and adaptation in kindergarten-aged children. J Aquat Phys Ther. 2006;14:8–17.

[11] Sigmundsson H, Hopkins B. Baby swimming: exploring the effects of early intervention on subsequent motor abilities. Child Care Health Dev. 2010;36:428–30. 19719766 10.1111/j.1365-2214.2009.00990.x

[12] Borioni F, Biino V, Tinagli V, Pesce C. Effects of baby swimming on motor and cognitive development: a pilot trial. Percept Mot Skills. 2022;129:977–1000. 35473471 10.1177/00315125221090203

[13] Leo I, Leone S, Dicataldo R, Vivenzio C, Cavallin N, Taglioni C, et al. A non-randomized pilot study on the benefits of baby swimming on motor development. Int J Environ Res Public Health. 2022;19: 9262. 35954617 10.3390/ijerph19159262 PMC9368508

[14] Jorge JAB, De J, Manoel E, Roberta RB, Okazaki VHA. Pilot study on infant swimming classes and early motor development. Percept Mot Skills. 2013;117:950–5. 24665810 10.2466/10.25.PMS.117x30z2

[15] Kawamoto T, Nitta H, Murata K, Toda E, Tsukamoto N, Hasegawa M, et al. Rationale and study design of the Japan environment and children’s study (JECS). BMC Public Health. 2014;14:1–8. 24410977 10.1186/1471-2458-14-25 PMC3893509

[16] Michikawa T, Nitta H, Nakayama SF, Yamazaki S, Isobe T, Tamura K, et al. Baseline profile of participants in the Japan environment and children’s study (JECS). J Epidemiol. 2018;28:99–104. 29093304 10.2188/jea.JE20170018 PMC5792233

[17] Iwai-Shimada M, Nakayama SF, Isobe T, Michikawa T, Yamazaki S, Nitta H, et al. Questionnaire results on exposure characteristics of pregnant women participating in the Japan Environment and Children Study (JECS). Environ Health Prev Med. 2018;23:1–15. 30219031 10.1186/s12199-018-0733-0 PMC6138908

[18] Bricker D, Squires J, Mounts L, Potter L, Nickel R, Twombly E, et al. Ages and stages questionnaires: a parent-completed child monitoring system. 3rd ed. Baltimore: Paul H Brookes Publishing Company; 2009.

[19] Mezawa H, Aoki S, Nakayama SF, Nitta H, Ikeda N, Kato K, et al. Psychometric profile of the ages and stages questionnaires, Japanese translation. Pediatr Int. 2019;61:1086–95. 31419360 10.1111/ped.13990 PMC6899956

[20] Mezawa H, Aoki S, Nakayama SF, Nitta H, Ikeda N, Kato K, et al. Psychometric profile of the ages and stages questionnaires, Japanese translation. Pediatr Int. 2019;61(11):1086–95. 31419360 10.1111/ped.13990 PMC6899956

[21] Gallahue DL, Ozmun JC. Understanding motor development: infants, children, adolescents, adults. 3rd ed. Madison: Brown & Benchmark; 1995.

[22] Malina RM. Motor development during infancy and early childhood: overview and suggested directions for research. Int J Sport Heal Sci. 2004;2:50–66.

[23] Hadders-Algra M. Early human motor development: from variation to the ability to vary and adapt. Neurosci Biobehav Rev. 2018;90:411–27. 29752957 10.1016/j.neubiorev.2018.05.009

[24] Goodway JD, Ozmun JC, Gallahue DL. Understanding motor development: infants, children, adolescents, adults. 8th ed. Burlington: Jones and Bartlett’s Learning; 2019.

[25] Scammon RE. The measurement of the body in childhood. In: Harris JA, Jackson CM, Paterson DG, Scammon RE, editors. The measurement of man. Minneapolis: University of Minnesota Press; 1930. p. 173–215.

[26] Klekamp J, Riedel A, Harper C, Kretschmann HJ. Quantitative changes during the postnatal maturation of the human visual cortex. J Neurol Sci. 1991;103:136–43. 1880530 10.1016/0022-510x(91)90156-2

[27] WHO Multicentre Growth Reference Study Group, de Onis M. WHO Motor Development Study: windows of achievement for six gross motor development milestones. Acta Paediatr Int J Paediatr. 2006;95:86–95. 10.1111/j.1651-2227.2006.tb02379.x 16817682

[28] Wijnhoven TMA, de Onis M, Onyango AW, Wang T, Bjoerneboe GEA, Bhandari N, et al. Asessment of gross motor development in the WHO Multicentre Growth Reference Study. Food Nutr Bull. 2004;25(1Suppl1):37–45. 10.1177/15648265040251S105 15069918

[29] Kimura-Ohba S, Sawada A, Shiotani Y, Matsuzawa S, Awaya T, Ikeda H, et al. Variations in early gross motor milestones and in the age of walking in Japanese children. Pediatr Int. 2011;53:950–5. 21752149 10.1111/j.1442-200X.2011.03423.x

[30] Largo RH, Molinari L, Weber M, Pinto LC, Duc G. Early development of locomotion: significance of prematurity, cerebral palsy and sex. Dev Med Child Neurol. 1985;27:183–91. 3996775 10.1111/j.1469-8749.1985.tb03768.x

[31] Meinel K, Schnabel G. Bewegungslehre-Sportmotorik. Berlin: Sport; 1998.

[32] Valeriani F, Protano C, Vitali M, Romano Spica V. Swimming attendance during childhood and development of asthma: meta-analysis. Pediatr Int. 2017;59:614–21. 28032933 10.1111/ped.13230

[33] Nystad W, Njå F, Magnus P, Nafstad P. Baby swimming increases the risk of recurrent respiratory tract infections and otitis media. Acta Paediatr Int J Paediatr. 2003;92:905–9. 10.1080/08035250310003587 12948064

[34] Costa MJ, Barbosa TM, Ramos A, Marinho DA. Effects of a swimming program on infants’ heart rate response. J Sports Med Phys Fitness. 2016;56:352–8. 25422869

